# Synergistic effects of temperature and light on photoprotection in the model diatom *Phaeodactylum tricornutum*

**DOI:** 10.1111/ppl.70039

**Published:** 2025-01-15

**Authors:** Chiara E. Giossi, Dila B. Bitnel, Marie A. Wünsch, Peter G. Kroth, Bernard Lepetit

**Affiliations:** ^1^ Department of Biology University of Konstanz Konstanz Germany; ^2^ Present address: Institute of Life Sciences, University of Rostock Rostock Germany

## Abstract

Diatoms dominate phytoplankton communities in turbulent waters, where light fluctuations can be frequent and intense. Due to this complex environment, these heterokont microalgae display remarkable photoprotection strategies, including a fast Non‐Photochemical Quenching (NPQ). However, in nature, several abiotic parameters (such as temperature) can influence the response of photosynthetic organisms to light stress in a synergistic or antagonistic manner. Yet, the combined effects of light and these other drivers on the photosynthetic and photoprotective capacity of diatoms are still poorly understood. In this work, we investigated the impact of short‐term temperature and light stress on the model diatom *Phaeodactylum tricornutum*, combining NPQ induction‐recovery assays or light curves with a broad gradient of superimposed temperature treatments (5 to 35°C). We employed mutant lines deficient in NPQ generation (*vde KO*) or recovery (*zep3 KO*) and wild type. We found that temperature and light have a synergistic effect: lower temperatures limited both the photosynthetic capacity and NPQ, while the general photophysiological performance was enhanced with warming, up to a heat‐stress limit (above 30°C). We discuss the temperature effects on NPQ induction and recovery and propose that these are independent from the energy requirements of the cells and result from altered xanthophyll cycle dynamics. Namely, we found that de‐epoxidation activity strongly increases with temperature, outweighing epoxidation and resulting in a positive increase of NPQ with temperature. Finally, we propose that in a short‐term time frame, temperature and light have a synergistic and not antagonistic effect, with a positive relationship between increasing temperature and NPQ.

## 1 INTRODUCTION

Diatoms are eukaryotic photosynthetic microorganisms that represent one of the major components of phytoplankton in our oceans, where they dominate nutrient‐rich turbulent waters characterised by sudden and extreme light changes (Field et al., 1998; Falkowski et al., 2004; Tozzi et al., 2004; Lavaud, 2007). While the fundamental processes of photosynthesis in diatoms correspond to those of all other oxygenic phototrophs, their photophysiology also presents some distinctive features (Lepetit et al., 2022). In most diatoms studied so far, the regulated energy dissipation known as Non‐Photochemical Quenching (NPQ) directly correlates with the thylakoid content of the xanthophyll pigment diatoxanthin (Goss et al., 2006; Blommaert et al., 2021). Indeed, contrarily to what happens in green algae and higher plants, the energy‐dependent component of NPQ (qE) in diatoms is directly controlled by the diadinoxanthin cycle, a specific form of xanthophyll cycling that involves the interconversion between only two pigments: diadinoxanthin and its de‐epoxidized form diatoxanthin. This interconversion is catalysed by the enzymes VIOLAXANTHIN DE‐EPOXIDASE (VDE) and ZEAXANTHIN EPOXIDASE (ZEP), regulated by a trans‐thylakoidal ΔpH (Goss and Lepetit, 2015). In addition, the qE capacity in diatoms relies on trans‐membrane antenna proteins of the Lhcx family (Buck et al., 2019; Croteau et al., 2024). Due to its strict dependency on diatoxanthin and Lhcx proteins, diatoms qE significantly differs from the process known in other photosynthetic eukaryotes and instead resembles the zeaxanthin‐dependent quenching of plants (Nilkens et al., 2010), albeit showing faster kinetics. Therefore, diatom qE is also referred to as qZ (Blommaert et al., 2021; Croteau et al., 2024). From this point, we will use either qE or NPQ to indicate this xanthophyll cycle‐dependent form of energy quenching.

In the oceans, additional drivers besides light (like temperature, pH, salinity, UV radiation or nutrient availability) can influence photosynthetic organisms and their photophysiology. Thus, multiple‐stressor studies are essential to understand how these organisms perform under natural conditions (Gunderson et al., 2016; Boyd et al., 2018; Gao et al., 2019; Wei et al., 2020). Indeed, due to the interaction of light with other environmental stress factors, in most global oceanic environments qE is thought to be constantly active to a certain extent (Lin et al., 2016).

Temperature has a strong and rapid impact on photosynthesis (Fracheboud and Leipner, 2003; Li et al., 2021). In aquatic ecosystems, temperature changes combined with light stress can alter the physiology and the structure of phytoplankton communities (Hancke et al., 2014; Lewandowska and Sommer, 2010; Zeng et al., 2020). Yet, the combined effects of temperature and light on qE and its related molecular partners are still poorly understood. Laviale et al. (2015) have shown that high temperatures combined with high light can limit (at least transiently) the photosynthetic activity of benthic diatoms communities in estuarine mudflats due to an enhancement of qE and related Lhcx proteins. Studies on polar diatoms (Ha et al., 2016; Lacour et al., 2018, 2022) have also shown that, in response to the rigid climate that they inhabit, these organisms display a strong NPQ capacity and maintain a constitutive pool of diatoxanthin, independent of light. However, while the combined effects of temperature and light on the growth and physiology of different diatom species have been investigated in detail (Fawley, 1984; Strzepek and Price, 2000; Zeng et al., 2020), studies characterizing the effects of a broad range of combined temperature and light stress on NPQ dynamics are still lacking.

In this work, we investigated the short‐term photophysiological effects of combined temperature and light stress on the model pennate diatom *Phaeodactylum tricornutum* acclimated to low light (30 μmol photons m^−2^ s^−1^) and mild temperature (20°C). We employed wild type (wt) and two control lines with altered qE capacity: knockouts of *VDE* and *ZEP3*, the main enzymes regulating xanthophyll cycling in this species (Giossi et al., 2024; Græsholt et al., 2024; Ware et al., 2024). We exposed our cultures to a broad temperature gradient ranging from 5 to 35°C (±15°C from the control) for about one hour, while also superimposing a light stress through NPQ induction‐recovery assays or light curve protocols. Through the analysis of different photophysiological parameters and xanthophyll cycle pigments content across temperatures, we discuss the thermal properties of the main enzymes regulating the xanthophyll cycle and how these influences the response to light stress in this diatom.

## 2 MATERIALS AND METHODS

### 2.1 Algal strains

For this study, we employed a wild type (wt) strain of the pennate diatom *Phaeodactylum tricornutum* (Pt1; CCAP1055) and corresponding CRISPR/Cas9 knockout lines for *VDE* [Phatr2_44635; chr_4:1116193–1117626(−)] (*vde KO*), and *ZEP3* [Phatr2_56492; chr_4:1118248–1120587(+)] (*zep3 KO*), recently generated in our laboratory (Giossi et al., 2024).

### 2.2 Culture conditions

*P. tricornutum* was grown in standard f/2 medium (Guillard, 1975) with 16 g/L of Tropic Marin CLASSIC sea salt (Dr. Biener GmbH) and without addition of silica. All strains were maintained for several months on f/2‐agar plates without antibiotic selection in a controlled growth chamber [16°C, ~10 μmol photons m^−2^ s^−1^ of white light provided by L 36 W/840 LUMILUX® Cool White tubes (OSRAM GmbH), with 16:8 h light:dark photoperiod]. All light intensities indicated in this work were measured in air with a spherical sensor (ULM‐500 light meter, Heinz Walz). Comparison with a planar sensor (LI‐185B Quantum Radiometer Photometer, LI‐COR Inc.) is presented in Table S1.

Fresh samples were inoculated in f/2 in glass Erlenmeyer flasks and grown in shaking batch cultures at 20°C, with about 30 μmol photons m^−2^ s^−1^ of white light (XT 58 W/830 LUMILUX® Warm White tubes, OSRAM GmbH) with 16:8 h light:dark photoperiod, until growth was visible by the naked eye (about 1 week). Then, cultures were moved to semi‐chemostatic conditions: cell density (determined with Multisizer 4e coulter counter, BECKMAN COULTER) was maintained in early exponential phase (0.5–2.5*10^6^ cells/mL) with regular addition of fresh f/2 medium for about 2 weeks. At the end of this acclimation phase, cultures were diluted to achieve a target concentration of 1–2*10^6^ cells/mL on the experimental day.

### 2.3 Experimental setup and PAM fluorescence analyses

To test the synergistic effect of temperature and light, we applied a recently published technique termed Phenoplate (Herdean et al., 2022), combining the use of an IMAGING‐PAM fluorometer (IMAG‐S module equipped with IMAG‐L LED‐Ring‐Array with blue light and IMAG‐K CCD‐camera, connected to an IMAG‐C control unit, Heinz Walz GmbH) and a PCR thermocycler (Mastercycler gradient 5331, Eppendorf) placed under the LED array of the fluorometer to simultaneously apply different combinations of temperature and light stress. Temperature was controlled using the “incubate” function of the thermocycler and the variables displayed in this manuscript correspond to the given input values (Herdean et al., 2022). Direct temperature measurements (TSUB21, FireSting‐O2, Pyroscience) in a representative sample yielded the input value ±3°C for all temperatures (Figure S1).

Exponentially grown cultures (1–2*10^6^ cells/mL) were transferred in a thermostatically controlled room (20°C, ambient light). Cultures were not dark adapted as this procedure is commonly avoided for diatoms due to the generation of NPQ in the absence of light, known as dark NPQ (Goss et al., 2006; Grouneva et al., 2009; Lepetit et al., 2013). One sample (250 μL) from each culture was inoculated in a white PCR well plate with addition of NaHCO_3_ (4 mM final concentration) to ensure sufficient CO_2_ supply throughout the experiment. The plate was immediately placed in the Phenoplate setup at 20°C (control temperature) with low‐intensity measuring light (ca. 1 Hz repetition rate). A first saturating pulse (3000 μmol photons m^−2^ s^−1^; width: 800 ms) was given in the dark to measure Fo and Fm (defined in Table 1), followed by 30 min of temperature treatment (defined below for each experiment) under low actinic blue light (28 μmol photons m^−2^ s^−1^). At the end of this step, light was switched off for about 30 s and a second saturating pulse in the dark was applied. This procedure was followed by either NPQ induction‐recovery assays or light curve experiments.

**TABLE 1:** Chlorophyll *a* fluorescence parameters.

Parameter	Definition
Fo and Fm	Minimum and maximum fluorescence yield in the dark
F and Fm′	Minimum and maximum fluorescence yield under actinic light
Fv/Fm = (Fm – Fo) / Fm	Quantum yield of Photosystem II in the dark
YII = (Fm′ – F) / Fm′	Quantum yield of Photosystem II under actinic light
NPQ = (Fm – Fm′) / Fm′	Non‐Photochemical Quenching
rETR = YII * PAR	Relative Electron Transport Rate
Ek	Light saturation coefficient of an rETR versus PAR curve
Eopt	Light intensity optimum of an rETR versus PAR curve, corresponding to the light intensity at which the maximum rETR is reached
NPQmax	Maximum NPQ in an NPQ versus PAR curve
E50	Irradiance level corresponding to 50% of NPQmax in an NPQ versus PAR curve

(Eilers and Peeters, 1988; Serôdio and Lavaud, 2011; Murchie and Lawson, 2013)

For NPQ induction‐recovery assays, performed independently at 5, 10, 15, 20, 25, 30 and 35°C, samples were treated for 5 min with strong actinic light (500 μmol photons m^−2^ s^−1^ blue light) followed by 15 min of recovery in low light (28 μmol photons m^−2^ s^−1^ blue light); saturating pulses were supplied every 20 s. To further investigate the detrimental effect of the highest temperature stress (35°C), two additional experiments with a shift between control temperature and heat stress between induction and recovery were performed (i.e., acclimation and induction at 20°C + recovery at 35°C versus acclimation and induction at 35°C + recovery at 20°C).

The same assays were repeated in the wt at all temperatures for pigment content analysis: 4 samples (each spread over 3 wells of 250 μL each, for a total of 750 μL/sample) were inoculated in the Phenoplate setup and sampled at the beginning of the experiment (at 20°C, before the temperature acclimation), after 30 min of temperature treatment (i.e., before the onset of the induction phase), at the end of the induction phase (i.e., after the 5 min of strong actinic light) or at the end of recovery phase (i.e., after the 15 min of low light). Due to the nature of the sampling, chlorophyll *a* fluorescence measurements were not acquired during this procedure; still, the same light protocol for NPQ induction‐recovery assays described above was supplied with the IMAGING‐PAM. Each sample (750 μL) was vacuum‐filtered (ISOPORE 1.2 μm 433 PC membrane filters, 25 mm), flash‐frozen in liquid nitrogen and stored at −80°C for subsequent HPLC analysis.

For light curves, performed independently at 5, 10, 15, 20, 25, and 30°C, samples were exposed to 12 increasing light steps (12, 28, 44, 60, 95, 129, 180, 232, 301, 388, 509, 645 μmol photons m^−2^ s^−1^ blue light) of 1 min each with a saturating pulse between each step, followed by 7.5 min of recovery in low light (28 μmol photons m^−2^ s^−1^) with 7 intermediate saturating pulses (after 60, 90, 120, 180, 240, 330 and 450 s).

Each experiment was repeated with biological triplicates (i.e., *n* = 3 cultures coming from time‐independent replications). More than one temperature treatment for each replicate was performed within the same day, starting at least 2 h after the beginning of the photoperiod, with a randomized sequence of treatments during each day.

### 2.4 Pigment extraction and HPLC analysis

Pigments were extracted from previously collected samples preserved at −80°C (described above). 650 μL of ice‐cold buffer (81% methanol, 9% 0.2 M ammonium acetate, 10% ethyl acetate) and a spoonful of glass beads were added to each sample, followed by vortexing (30s) and centrifugation at 4°C for 2 min at max speed (Jakob et al., 1999).

The supernatant was processed through a NUCLEOSIL® C18 column (EC 250/4, 300‐5, MACHEREY‐NAGEL GmbH) in a LaChrome Elite HPLC equipped with L‐2130 pump module, L‐2455 diode array detector, and L‐2200 autosampler (VWR International). Injected samples (80 μL) were separated with the three‐phase gradient described by Kraay et al. (1992) (eluent A: 85% methanol, 15% 0.5 M ammonium acetate in water; B: 90% acetonitrile, 10% water; C: 100% ethyl acetate), with 0.8 mL/min flow rate and a 35 min elution programme. Pigment identification was performed manually, based on known absorption spectra and retention times (Roy et al., 2011). Final concentrations (pmol/mL) were calculated using peak areas at 440 nm, integrated with the EZChrome Elite software (Agilent), and previously established calibration factors.

### 2.5 Determination of Lhcx content

Temperature‐driven changes in the amount of Lhcx proteins were assessed with western blots. Wt cultures were inoculated in f/2 in Erlenmeyer flasks in a controlled growth chamber (20°C, 30 μmol photons m^−2^ s^−1^ of white light provided by LEDs), with 16:8 h light:dark photoperiod and grown exponentially (<3*10^6^ cells/mL) with shaking and regular addition of fresh f/2 medium for about 2 weeks.

On the day of the experiment, cultures were adjusted to 2*10^6^ cells/mL with f/2 medium and shifted to an IS‐K Incubator (Noctua). Light treatment followed the approach described for NPQ induction recovery assays, except that for the 5 min of strong actinic light exposure ~700 μmol photons m^−2^ s^−1^ of white light (Philips Tornado and Bioledex LEDs) were provided to account for the stronger light attenuation in the Erlenmayer flask due to the larger algal culture volume needed. Cultures were briefly incubated at 20°C, and then shifted to 10°C, 20°C or 30°C for the rest of the experiment. After 50 min, temperature measured within the culture yielded 15°C for the 10°C treatment, and 28°C for the 30°C. Cells (200 mL) were harvested by filtration on ISPORE Polycarbonate 1.2‐mm filters (Millipore) and immediately frozen in liquid nitrogen at three time points: before the temperature shift, after 30 min incubation in low light at the new temperature, and after 20 min of induction phase (5 min) followed by recovery (15 min).

Protein Isolation and Western‐Blots were performed as described in Buck et al. (2021) with the following adjustments: Anti‐Lhcx (1:5000) and Anti‐RbcL (1:10000, serving as loading control) were incubated for 1,5 h, followed by incubation with the secondary antibody (1:10000 and 1:20000) for 1 h. Chemiluminescence signals were detected using Roti‐Lumin Plus (Carl Roth) with ECL Chemostar System (INTAS Science Imaging Instruments GmbH).

### 2.6 Data analysis and statistics

All chlorophyll *a* fluorescence parameters measured and calculated in this study are defined in Table 1. Relative Electron Transport Rate (rETR) versus Photosynthetically Active Radiation (PAR) curves were fitted with the model from Eilers and Peeters (1988), while NPQ versus PAR curves according to Serôdio and Lavaud (2011). We tested for statistical significance differences using Linear Mixed‐Effect Model (LMM, computing p‐values from the t‐statistics using Satterthwaite's approximation for degrees of freedom), multiple comparison t‐test or pairwise t‐test, depending on the data. The analysis was performed with R 4.2.1 (R foundation for statistical computing, Vienna, Austria) and a detailed script is available as Appendix S4.

## 3 RESULTS

### 3.1 NPQ induction‐recovery assays and investigation of the upper temperature stress limit

To explore the short‐term effects of combined temperature and light stress on the photophysiology of *P. tricornutum*, we employed a wt strain and two NPQ‐related mutant lines: *vde KO*, which is unable to induce qE due to lack of VDE, and *zep3 KO*, which is lacking ZEP3 and thus is unable to rapidly tune down NPQ after light stress (Giossi et al., 2024).

**FIGURE 1:**
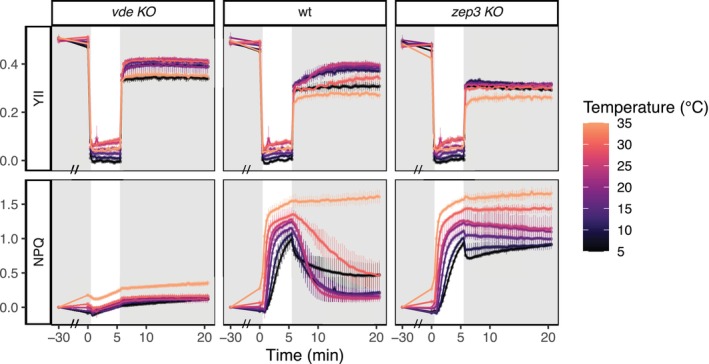
**YII and NPQ of** ***vde KO*** **, wt and** ***zep3 KO*** **during NPQ induction‐recovery assays performed under different temperature stress (average ± sd, n = 3)**. Samples were exposed for 30 min to low light (28 μmol photons m^−2^ s^−1^, highlighted in gray) to the corresponding temperature stress, followed by 5 min of NPQ induction (500 μmol photons m^−2^ s^−1^) and 15 min of recovery (28 μmol photons m^−2^ s^−1^, highlighted in gray) at the same temperature. On the x‐axis, time 0 indicates the beginning of the induction phase; an axis break, indicated by a double dash (//), was inserted during the first 30 min of temperature treatment (i.e., between −30 and 0 min). Results from the corresponding statistical analysis are presented in Table S2.

We first performed NPQ induction‐recovery assays with a broad range of temperatures (5–35°C), spanning from 20°C as control: samples were first pretreated with temperature for 30 min in low light, followed by 5 min of NPQ induction under light stress and 15 min of recovery in low light (Figure 1). Temperature alone did not have a massive effect on the quantum yield of photosystem II (YII): the first 30 min of treatment in low light (from −30 to 0 min) did not affect YII in all strains at any of the investigated temperatures, except for a slight decrease at the upper (35°C) and lower (5°C) temperature limit. This decrease was statistically significant in the wt and in the *zep3 KO*, but not in the *vde KO*. Contextually, all cultures showed a significant NPQ increase at 35°C during the temperature acclimation phase.

Temperature affected the photoprotective response to light stress of all our strains: NPQ was induced more rapidly at higher temperatures (25–35°C) in both wt and *zep3 KO*, reaching a plateau after the first 2 min of induction, while this was hardly reached after 5 min of induction at lower temperatures (5–15°C). Moreover, the NPQ value reached after 5 min of induction increased positively with temperature in both lines. As expected, no NPQ was detected in the *vde KO*. NPQ recovery in the wt was best around the control temperature (20 ± 5°C) but was progressively hindered in both lower (10–5°C) and higher temperatures (30–35°C). Furthermore, NPQ recovery was completely inhibited at 35°C in the wt: NPQ traces were identical to those of the *zep3 KO*. Interestingly, *vde KO* cells displayed an almost linear increase of NPQ at 35°C, which was also visible during the recovery phase of *zep3 KO* and wt. This most likely corresponds to the photoinhibitory component of NPQ (qI). At the lowest temperatures (10–5°C), NPQ showed an abrupt decrease during the first seconds of recovery. This pattern was particularly visible in the *zep3 KO*, indicating that this effect is independent of xanthophyll cycling.

**FIGURE 2:**
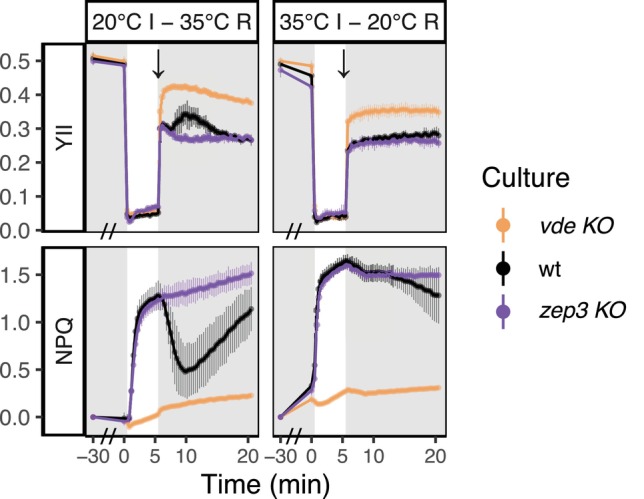
**YII and NPQ of** ***vde KO*** **, wt and** ***zep3 KO*** **during NPQ induction‐recovery assays performed with a temperature switch between induction (I) and recovery (R) (average ± sd, n = 3)**. Samples were exposed for 30 min to low light (28 μmol photons m^−2^ s^−1^, highlighted in gray) followed by 5 min of induction (500 μmol photons m^−2^ s^−1^) at the corresponding induction temperature (indicated on top of the panels) and 15 min of recovery (28 μmol photons m^−2^ s^−1^, highlighted in gray) at the corresponding recovery temperature (indicated on top of the panels). Temperature was switched at the end of the induction phase, indicated by a black arrow (↓). On the x‐axis, time 0 indicates the beginning of the induction phase; an axis break, indicated by a double dash (//), was inserted during the first 30 min of temperature treatment (i.e., between −30 and 0 min).

To further investigate the effects of higher temperatures on NPQ dynamics and assess the presence of a heat stress threshold, we conducted modified NPQ induction‐recovery assays with a switch of temperature between induction and recovery phase: cultures were exposed for 30 min to low light followed by 5 min of induction at either 20°C (control) or 35°C (heat stress); then the temperature treatment was switched (from control to 35°C and vice versa) for the remaining 15 min of recovery in low light (Figure 2). When the temperature stress (35°C) was introduced only during recovery, NPQ was initially tuned down in the wt; however, after the first 5 min of recovery all strains showed a linear increase of NPQ, together with a decrease of YII. On the other hand, when pre‐acclimation and induction phases were performed at 35°C, followed by recovery at 20°C, wt samples were not able to rapidly tune down NPQ during the first minutes of recovery, but began to slightly relax only after about 10 min of low light. These results, coherently with what is shown in Figure 1, suggest that temperatures above 30°C induce a generalised heat stress in *P. tricornutum*.

### 3.2 Analysis of xanthophyll cycle pigments content

**FIGURE 3:**
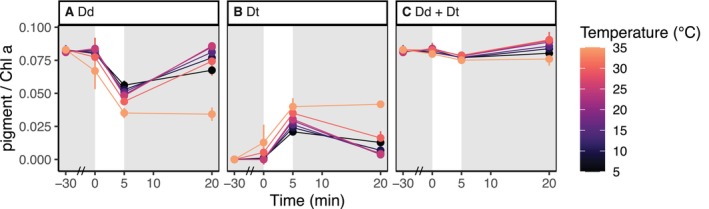
**Content of diadinoxanthin cycle pigments in wt exposed to different temperatures during NPQ induction‐recovery assays (average ± sd, n = 3)**. Pigment content is expressed as pigment:chlorophyll *a* ratio (mol/mol). Samples were exposed for 30 min in low light (28 μmol photons m^−2^ s^−1^, highlighted in gray) to the corresponding temperature, following 5 min of induction (500 μmol photons m^−2^ s^−1^) and 15 min of recovery (28 μmol photons m^−2^ s^−1^, highlighted in gray) under the same temperature. On the x‐axis, time 0 indicates the beginning of the induction phase; an axis break, indicated by a double dash (//), was inserted during the first 30 min of temperature treatment (i.e., between −30 and 0 min). (**A**) diadinoxanthin (Dd); (**B**) diatoxanthin (Dt); (**C**) total diadinoxanthin cycle pool (Dd + Dt). Results from the corresponding statistical analysis are presented in Table S3.

To investigate if the changes in NPQ performance under different temperatures were caused by altered xanthophyll cycle dynamics, we repeated the NPQ induction‐recovery assays with the wt and quantified the respective pigments. Samples were harvested before the experiment (−30 min), at the beginning (0 min) and end (5 min) of the induction phase, and at the end of the recovery phase (20 min). As shown in Figure 3, diatoxanthin accumulation during light induction increased gradually with temperature (from 5 to 35°C). In the recovery phase, diatoxanthin epoxidation was less efficient (i.e., a higher amount of diatoxanthin was present after 15 min of recovery) at both low (5°C) and high (30°C) temperatures, and was completely inhibited at 35°C. Moreover, at higher temperatures (35°C), small amounts of diatoxanthin were already detected during the first 30 min of temperature acclimation, indicating that diadinoxanthin de‐epoxidation was already active under low light. These results are in line with the NPQ data (Figure 1).

### 3.3 Light curves: analysis of rETR and NPQ versus PAR curves under different temperatures

**FIGURE 4:**
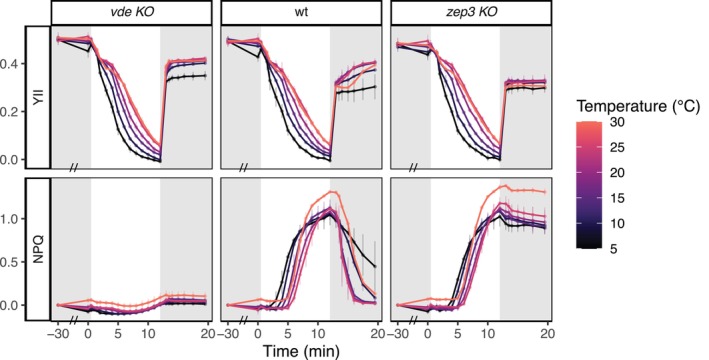
**YII and NPQ development over time in** ***vde KO*** **, wt and** ***zep3 KO*** **during light curve experiments performed under different temperature stress (average ± sd, n = 3)**. Samples were exposed for 30 min in low light (28 μmol photons m^−2^ s^−1^, highlighted in gray) to the corresponding temperature stress, followed by 12 light curve steps (12–645 μmol photons m^−2^ s^−1^) and 7.5 min of recovery (28 μmol photons m^−2^ s^−1^, highlighted in gray) under the same temperature. On the x‐axis, time 0 indicates the beginning of the induction phase; an axis break, indicated by a double dash (//), was inserted during the first 30 min of temperature treatment (i.e., between −30 and 0 min). Results from the corresponding statistical analysis are presented in Table S4.

After concluding that temperatures above 30°C induce a generalised heat stress, we performed light curves experiments with temperatures ranging from 5 to 30°C. For all lines, we observed a significant decrease of YII at colder temperatures (Figure 4). Contextually, in both wt and *zep3 KO*, the onset of NPQ was progressively retarded with increasing temperature, albeit reaching significantly higher values at 30°C. Recovery followed the same pattern observed during the previous experiments (Figure 1): NPQ recovery was impaired both at warmer (30°C) and colder (5°C) temperatures, indicating that the optimal recovery performance is achieved around the control temperature (20°C).

**FIGURE 5:**
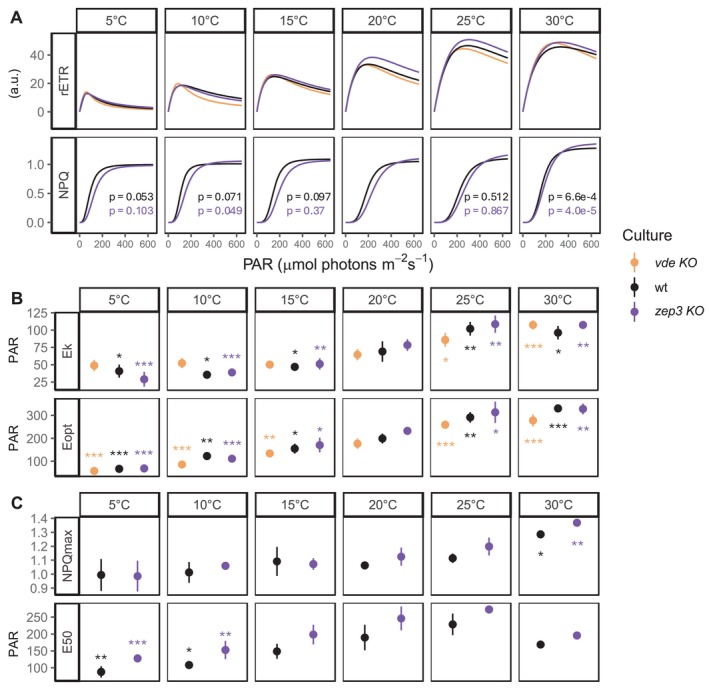
**Fitted rETR and NPQ versus PAR curves of** ***vde KO*** **, wt and** ***zep3 KO*** **during light curve experiments performed under different temperature stress**. After 30 min of temperature treatment (indicated on top of the panels) in low light (28 μmol photons m^−2^ s^−1^), samples were treated with 12 increasing steps of actinic light (12–645 μmol photons m^−2^ s^−1^) under the same temperature. (**A**) Curves fit. Hollow points represent average values (± sd, n = 3), while solid lines represent the curve fit obtained from all single replicate values (n = 3). Absence of line indicates that fitting was not possible. p‐values (p) indicate the statistical significance of differences between fitted curves at a given temperature and the reference (20°C), according to pairwise t‐test. Differences in rETR were not tested as this parameter carries a.u. (**B**) rETR curve parameters: Ek and Eopt. (**C**) NPQ curve parameters: NPQmax and E50; *vde KO* was excluded from this analysis as these lines always displayed values of NPQ close to 0, determining very poor model fitting. Points represent average values (± sd, n = 3) of curves fitted separately for each replicate. For each time point and within each culture, asterisks indicate the statistical significance of each temperature condition tested against the control (20°C), according to adjusted p‐value of multiple comparison t‐test: *: *p* < 0.05; **: *p* < 0.005, *** *p* < 0.005.

To better elucidate these dynamics, we analysed the light curve phase of our experiment (0–12 min) and fitted rETR and NPQ versus PAR curves with models from Eilers and Peeters (1988) and Serôdio and Lavaud (2011). We here defined rETR as YII*PAR (Table 1) (Serôdio and Lavaud, 2011). This parameter does not account for the quanta distribution between photosystem and the absorptivity of the culture and is thus expressed in arbitrary units (a.u.). While this rETR should not be regarded as absolute ETR or used for quantitative comparison with other independent studies, the corresponding rETR/PAR curves provide a reliable and robust tool to assess differences in the light use efficiency across different strains and treatments within the same experiment, as performed in the present study. rETR/PAR curves (Figure 5) revealed that the photosynthetic efficiency generally increased with temperature: both Ek and Eopt decreased significantly from control (20°C) to colder temperatures (15–5°C), and increased significantly in response to higher temperatures (25–30°C). This trend was observed in all cultures, with the exception of the absence of significant decrease of Ek at colder temperatures in the *vde KO*.

NPQ versus PAR curves (Figure 5) revealed a positive increase of NPQ and NPQmax with temperature for both wt and *zep3 KO* lines, with a significant increase of both parameters at 30°C. Contextually, E50 also gradually increased from 5°C towards the control, indicating that sustained energy dissipation was already reached at lower light intensities when the cells were exposed to lower temperatures. We also reported a shift in the lower light limit for NPQ induction from low to high temperatures: at 5°C NPQ was already induced at lower light intensities (44 μmol photons m^−2^ s^−1^) in both wt and *zep3 KO*, while YII rapidly decreased and reached 0 at the strongest light step (645 μmol photons m^−2^ s^−1^). This suggests that cold treatment reduced the photosynthetic rate of the cultures and, therefore, determined the need for energy quenching at lower light intensities. Quenching activation was then progressively delayed with increasing temperature: at higher temperatures (20–25°C) NPQ was tuned on only at higher light intensities. At 30°C, the cultures displayed the highest NPQmax. This indicates the activation of stronger energy quenching, supported by the production of small but significant NPQ generation during the first 30 min of low light at this temperature (Figure 4).

## 4 DISCUSSION

### 4.1 Short‐term cold treatment affects the light‐use efficiency of *P. tricornutum* under light stress and slows down NPQ induction and recovery

Exposure to lower temperatures has almost no direct effect on the light reactions of photosynthesis, but negatively affects the carbon assimilation rate and increases the excitation pressure on Photosystem II due to less efficient energy consumption (Huner et al., 1998; Fracheboud and Leipner, 2003). As a consequence, chilling can increase the demand for thermal energy dissipation via xanthophyll‐related qE (Bilger and Björkman, 1991; Adams and Barker, 1998; Ni et al., 2017) while slowing down NPQ recovery (Verhoeven et al., 1998). Coherently, lower temperatures alone did not cause a drastic reduction of YII in our experiments (Figures 1 and 4) but altered the cellular response to light stress: in *P. tricornutum* cells exposed to colder temperatures (5–15°C), photosynthesis saturated much earlier and NPQ was induced at lower light intensities (Figure 5). Quenching activation was also slower at lower temperatures: following induction with a fixed light intensity (Figure 1), cultures exposed to 5°C did not achieve complete NPQ induction as rapidly and intensely as at higher temperatures. Moreover, although during light curve experiments (Figures 4, 5) cultures exposed to lower temperatures tuned on NPQ at lower light intensities, E50 significantly decreased from 20 to 5°C (Figure 5), indicating a reduced quenching induction in the cold.

A sustained NPQ is essential for diatoms adapted to cold environments (Ha et al., 2016; Lacour et al., 2018, 2022). In the framework of our short‐term study, lower temperatures had negative effects on both NPQ induction and recovery, despite the photosynthetic efficiency of the cultures being reduced. While these results can seem contradictory, they highlight the differences between prolonged acclimation or adaptation to lower temperatures and the immediate effect of abrupt chilling. Diatoms can efficiently adjust their gene expression and photosynthetic machinery to compensate for temperature‐driven physiological changes (Fanesi et al., 2016; Liang et al., 2019). However, such tuning of the photoprotective machinery requires longer acclimation times, as it usually involves new protein synthesis and lipid remodelling (Wu et al., 2022; Shomo et al., 2024). Another sensitive indicator of long‐term acclimation to new abiotic conditions is the xanthophyll cycle pool size (Petrou et al., 2011; Lepetit et al., 2017; Lacour et al., 2020; Buck et al., 2022), which did not change significantly during our experiment in the wt (Figure 3C). We, therefore, conclude that the impaired NPQ dynamics we observed are connected to the short‐term effects of sub‐optimal temperatures on the molecular mechanisms that regulate quenching and xanthophyll cycling.

### 4.2 *zep3 KO* and NPQ recovery in the cold: a new approach to investigate the relationship between pH and energy quenching

With the lowest temperature treatments (10–5°C), we observed a sudden and sharp NPQ decrease at the beginning of the recovery phase in wt and *zep3 KO* lines (Figure 1). In *P. tricornutum*, NPQ and diatoxanthin have a well‐known linear dependency (Goss et al., 2006; Blommaert et al., 2021; Croteau et al., 2024). Thus, as ZEP3‐deficient mutants cannot rapidly epoxidize diatoxanthin (Giossi et al., 2024; Græsholt et al., 2024; Ware et al., 2024), the sudden recovery of NPQ in the cold we observed violates the canonical Stern Volmer relationship between diatoxanthin and quenching. Similar violations have also been observed under other conditions (Lavaud and Kroth, 2006; Croteau et al., 2024), but this phenomenon remains so far unexplained. Several hypotheses regarding a pH‐dependent component of NPQ in diatoms have been raised, but the existence of a prominent diatoxanthin‐independent quenching (at least in terms of qE/qZ) has been experimentally ruled out (Blommaert et al., 2021; Croteau et al., 2024; Giossi et al., 2024). However, Daskalakis et al. (2024) have recently shown that, under low pH, a water channel might be formed within the main light‐harvesting antenna of diatoms, leading to a chlorophyll protonation that is potentially important for NPQ generation, thus raising new arguments on the relationship between energy quenching and pH. While such questions are out of the scope of this study, we propose that the combination of ZEP3‐deficient *P. tricornutum* and various temperature treatments could be a suitable system to investigate the mechanistic principles underlying this exceptional deviation of the linear relationship between diatoxanthin and NPQ in diatoms.

### 4.3 Warming enhanced the photophysiological performance of *P. tricornutum* up to a heat stress limit

Due to the pressing challenges of climate change (IPCC, 2023), the effects of increasing temperatures on the ecology of an essential component of phytoplankton, like diatoms, have been gaining increasing attention. From a photophysiological point of view, warming positively influences all enzymatic kinetics, including those related to photosynthetic reactions, but heat stress can ultimately lead to inhibition of important enzymes (like Photosystem II or Rubisco), formation of reactive oxygen species and photoinhibition (Mathur et al., 2014). Moreover, higher temperatures favour the oxygenase reaction of Rubisco over the carboxylation one. Experimental evidence has indeed shown that higher temperatures can increase photosynthetic rates, growth and carbon uptake of diatoms, and even enhance their photoprotection capacity against UV, but can also lead to negative outcomes like higher antioxidant stress and reduced biomass (Feijão et al., 2018; Zeng et al., 2020; Duarte Moreno et al., 2023; Rehder et al., 2023; Xu et al., 2023). In two separate studies on diatoms‐dominated microphytobenthos communities, warming had a positive effect on the photosynthetic capacity, but also increased photoinhibition at really high temperatures (35°C) (Vieira et al., 2013; Laviale et al., 2015), stressing the importance of identifying the boundary between thermal enhancement and heat stress.

**FIGURE 6:**
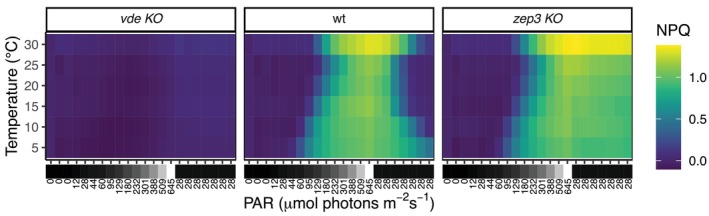
**Under short time frames, temperature and light have a synergistic effect on NPQ in** ***P. tricornutum***. Summary heatmap with the results of our light curve experiment (average NPQ, n = 3). On the × axis, each line represents a temperature treatment. On the y axis, each column represents a single sample (ordered according to sampling time); corresponding light intensity (PAR) is plotted below the × axis.

In our work, we concluded that temperatures above 30°C induce heat stress in *P. tricornutum*: at 35°C, cultures were not able to relax NPQ (Figures 1 and 2) and the corresponding diatoxanthin pool (Figure 3). *vde KO* also showed a linear increase of NPQ at 35°C. As this line essentially lacks qE, this quantifiable NPQ increase results from a decrease of fluorescence likely due to generalised heat stress, and thus represents the contribution of the photoinhibitory quenching (qI) and not active qE induction. Below this limit, temperatures between 25 and 30°C enhanced the photosynthetic performance and determined a faster NPQ induction and recovery (Figures 1 and 4, 5, 6). Early studies had also reported enhanced photosynthetic carbon assimilation in *P. tricornutum* exposed to temperatures higher than its growing conditions, up to a 35°C limit (William and Morris, 1982). Moreover, during our light curve experiments (Figures 4, 5, 6), the onset of NPQ was shifted to higher light intensities under warmer temperatures but rapidly reached high NPQmax values. In accordance with previous observations (Li et al., 2021), these results suggest that, until a threshold of heat stress is reached, the photosynthetic apparatus of *P. tricornutum* can efficiently respond to the temperature increase, displaying a better use of light energy (e.g., due to enhanced carbon assimilation operated by Rubisco) but also employing a fast and strong NPQ capacity.

### 4.4 Dynamics of NPQ induction and recovery across temperatures indicate different thermal properties of VDE and ZEP3

Due to its strict relationship with the enzymatic de‐epoxidation of diadinoxanthin to diatoxanthin, qE generation in diatoms will depend mostly on the enzymatic kinetics of VDE and ZEP3 if the Lhcx pool remains constant (Blommaert et al., 2021; Croteau et al., 2024; Giossi et al., 2024). In the framework of our experiments, the Lhcx pool is not likely to be altered significantly: during severe high light stress, it takes more than 30 min until a significant amount of Lhcx proteins is newly synthesised (Lepetit et al., 2013). Moreover, western blots showed no difference in Lhcx content between cultures exposed to 10, 20 or 30°C for 50 min in an independent experiment comparable to our NPQ induction‐relaxation assays (Figure S2). We thus conclude that the alterations of NPQ induction and recovery we observed can be largely attributed to changes in the activity of VDE and ZEP3. Namely, temperature clearly had a different effect on NPQ induction and recovery: while in all our experiments induction increased with warming, recovery was hindered both by lower and higher temperatures.

An increase in de‐epoxidation activity with temperatures has been reported in different plants and diatoms species (Bilger and Björkman, 1991; Latowski et al., 2003; Zhang et al., 2009; Salleh and McMinn, 2011; Chen and Gallie, 2012), also when coupled with high light (Laviale et al., 2015). In plants, algae and *in vitro* systems, the kinetic de‐epoxidation is also influenced by membrane fluidity, which is greatly affected by temperature changes (Goss and Latowski, 2020). Moreover, diatoms VDE has been proven to be essential for the short‐term temperature response due to the thylakoid membrane stabilising function of diatoxanthin (Bojko et al., 2019). Therefore, a positive relationship between VDE activity and temperature, independent from the energy requirements of the cell, may easily explain the fast and intense NPQ induction we observed with increasing temperatures in *P. tricornutum*. In addition, high temperatures can increase chlororespiration (Quiles, 2006), a process that has been described in diatoms in the dark (Jakob et al., 1999; Jakob et al., 2001). While it is meanwhile assumed that the actual mechanistic identity of “chlororespiration” in diatoms is rather a reverse activity of the plastidic ATP synthase (Bailleul et al. 2015; Lepetit et al. 2022), such a process would contribute to the acidification of the thylakoid lumen and thus increase VDE activity and the consequent formation of diatoxanthin. In plants, warming can negatively affect qE due to a disruption of the ΔpH gradient that is only partially compensated by enhanced VDE activity (Zhang et al., 2009). However, diatoms lack a prominent ΔpH‐dependent component of quenching, and their VDE is activated at a much higher pH compared to plants (Goss et al., 2006; Jakob et al., 2001). These characteristic features likely explain why we observed a quite steady increase of NPQ with higher temperatures, paralleled with diatoxanthin accumulation.

Recovery, on the other hand, was optimal only around the control temperature (i.e., 20°C, the condition to which the organism was acclimated), meaning that diatoxanthin epoxidation activity is affected both by low‐ and high‐temperature stress. Plants exposed to cold display slower NPQ recovery, meaning an impaired ZEP activity (Verhoeven et al., 1998; Reinhold et al., 2008) that seems to be essential for alleviating Photosystem II photoinhibition under high light and chilling stress (Wang et al., 2008). A similar process happens with warming (Salleh and McMinn, 2011). Laviale et al. (2015) proposed that a slower recovery, observed in natural assemblages of benthic diatoms exposed to high temperature and high light, enhances the photoprotection capacity and possibly represents a feedback response to the impairment of the downstream reactions of photosynthesis under extreme stress conditions. Based on our results, we propose that, while the impairment of NPQ recovery is most likely due to a reduction of ZEP3 kinetics under cold temperatures, the suboptimal recovery under higher temperatures is likely due to the counteracting effect of an enhanced VDE. A similar relationship between VDE and ZEP has also been reported in plants (Zhang et al., 2011). We propose that this could rise from diverging thermal properties of the two enzymes or from changes in substrate access and/or binding under different temperatures.

Notably, in experiments performed with a temperature change between induction and recovery phase (Figure 2), the introduction of high‐temperature stress (35°C) after the light stress (i.e., during recovery in low light) caused not only the activation of NPQ in the wt but also in the *zep3 KO* (Figure 2). Although we reported a slight increase in the measured NPQ (likely corresponding to qI) in both *vde KO* and *zep3 KO* under constant 35°C stress (Figure 1), this was clearly stronger in the *zep3 KO* during the temperature‐switch experiment (Figure 2). We propose that this is most likely due to an additional diatoxanthin accumulation due to an increased VDE activity, triggered by thermal enhancement and/or by an increase of chlororespiration/reverse activity of the ATP synthase and consequent acidification of the thylakoid lumen. Alternatively, accumulation of diatoxanthin could also be triggered by thermal inactivation of the epoxidase. Our pigment analysis (Figure 3) also indicates that warming alone is sufficient to trigger diatoxanthin accumulation in the wt (i.e., under low light).

All together, these results stress the importance of the regulation of the equilibrium between epoxidation and de‐epoxidation activity in regulating diatoms NPQ. Indeed, Blommaert et al. (2021) had previously proposed that the balance between ZEP and VDE is key for activation and regulation of NPQ in *P. tricornutum*. Here, we demonstrated that this holds true also when light is coupled with temperature stress.

## 5 CONCLUSION

Temperature and light have a fundamental impact on the energy balance of photosynthetic cells. Several studies have proposed that these factors have antagonistic effects on phytoplankton photophysiology (Helbling et al., 2011; Fanesi et al., 2016; Zeng et al., 2020; Li et al., 2021). On one hand, both high light and low temperature increase the demand for energy quenching due to an energy surplus caused by an excessive photons influx (high light) or by inefficient energy use (low temperature). Conversely, low light and high temperature cause an energy deficit with respect to the cell metabolic demand, resulting in lower energy dissipation requirements. For this reason, we originally hypothesised that combined stress should either mitigate or enhance the effects of light on NPQ in *P. tricornutum*. Namely, a combination of high light and low temperature would cause an even more drastic NPQ increase, while energy quenching should be the lowest under high temperature and low light.

Our study revealed a more complex outcome (Figure 6). In the short‐time framework of our experiments, temperature had a more drastic impact on the dynamics of NPQ and xanthophyll cycling of *P. tricornutum*: high temperatures had the strongest NPQ response when coupled with high light. Conversely, while the photoprotective response was activated already at lower irradiances in colder temperatures, this condition limited the intensity of the NPQ response due to less efficient diatoxanthin accumulation. We suggest that, on a short‐term time frame, temperature increase has a decisive impact on photoprotection dynamics and exacerbates the effects of high light instead of mitigating it. We propose that the positive increase of NPQ capacity with temperatures is not directly related with photosynthesis requirements, and instead depends on the molecular dynamics of xanthophyll cycling of *P. tricornutum*.

## AUTHOR CONTRIBUTIONS

C.E.G and B.L. designed the project; C.E.G., B.L. and D.B.B. designed experiments; C.E.G and M.A.W. performed preliminary work (e.g., generation of mutant lines and experiments excluded from the final study); D.B.B. performed and analysed preliminary experiments under supervision of C.E.G., B.L. and P.G.K.; C.E.G. performed the final experiments and the data analysis presented in this manuscript, supervised by B.L.; M.A.W. performed the western blots for determination of *Lhcx1* content and the related experiments; C.E.G. wrote the initial draft of the manuscript, edited by B.L; The manuscript was revised and completed with contribution of all authors.

## FUNDING INFORMATION

B.L. received funding from DFG (German Research Foundation, Bonn, Germany): LE 3358/3–2 and Heisenberg program. C.E.G. acknowledges support from Studienstiftung des Deutschen Volkes (Bonn, Germany) and LGFG (Act on Graduate Funding of the State of Baden‐Württemberg, University of Konstanz, Germany). P.G.K. acknowledges support by the University of Konstanz.

## Supporting information


**Appendix S1:** Raw PAM data generated for the NPQ induction‐recovery assays (Fig. 1‐2). In order, columns represent: file_index: unique number assigned to each original data file; Date: date of the experiment; Time: relative sampling time (s); Temperature: temperature variable in°C indicating standard assays (5‐35) or the assays with temperature switch (“20 to 35” or “35 to 20”); Replicate: unique text assigned to each replicate; pulseN: number automatically assigned to each pulse; PAR: corresponding PAR in μmol photons m‐2 s‐1; Time_abs: absolute sampling time (ms); F: Fo or F; Fm': Fm or Fm'; Culture: unique text assigned to each culture (wt = wt; zep3 = zep3 KO; vde = vde KO).

**Appendix S2:** Raw PAM data generated for the light curve assays (Fig. 4‐6). In order, columns represent: file_index: unique number assigned to each original data file; Date: date of the experiment; Time: relative sampling time (s); Temperature: temperature variable in°C; Replicate: unique text assigned to each replicate; pulseN: number automatically assigned to each pulse; PAR: corresponding PAR in μmol photons m‐2 s‐1; Time_abs: absolute sampling time (ms); F: Fo or F; Fm': Fm or Fm'; Culture: unique name assigned to each culture (wt = wt; zep3 = zep3 KO; vde = vde KO).

**Appendix S3:** Raw pigment data (Fig. 3). In order, columns represent: Replicate: unique text assigned to each replicate; Culture: unique name assigned to each culture (wt = wt; zep3 = zep3 KO; vde = vde KO); Time: relative sampling time (min); Vhar: sample volume harvested (mL); Vext: volume of extraction buffer (mL); Vinj: volume injected (mL); Chlide: chlorophyllide a; Chlc: chlorophyll c; Fx: fucoxanthin; Fxi: fucoxanthin‐isomer; Vx: violaxanthin; Dd: diadinoxanthin; Ax: antheraxanthin; Dt: diatoxanthin; Chla: chlorophyll a; Bcar: β‐carotene; cBcar: cis‐β‐carotene. Pigment content is expressed in pmol/mL. Empty cells indicate that the corresponding pigment was not detected.

**Appendix S4:** Detailed R script containing the data analysis and statistics used in this study.

**Appendix S5:** supporting Information

## Data Availability

The data that supports the findings of this study are available in the supplementary material of this article (Appendixes [Link], [Link]). Mutant algal strains are cryopreserved in the laboratory of P.G.K. and are available upon request. Additional queries can be addressed to the corresponding authors.
