# Supplementary material for: Synergistic effects of temperature and light on photoprotection in the model diatom Phaeodactylum tricornutum
Source: Physiol Plant. 2025 Jan 15;177(1):e70039. doi: 10.1111/ppl.70039 (PMC11733657; doi:10.1111/ppl.70039)
Supplement: Supplementary file 5 — Appendix S5: supporting Information [file PPL-177-e70039-s005.pdf]

Supporting information for:

Synergistic effects of temperature and light on photoprotection in the model diatom *Phaeodactylum tricornutum*

Chiara E. Giossi[a*], Dila B. Bitnel[a], Marie A. Wünsch[a,b], Peter G. Kroth[a], Bernard Lepetit[a,b*]

[a] Department of Biology, University of Konstanz, 78464 Konstanz, Germany
[b] Present address: Institute of Life Sciences, University of Rostock, 18059 Rostock, Germany

***To whom correspondence may be addressed:**
chiara.giossi@uni-konstanz.de, bernard.lepetit@uni-rostock.de

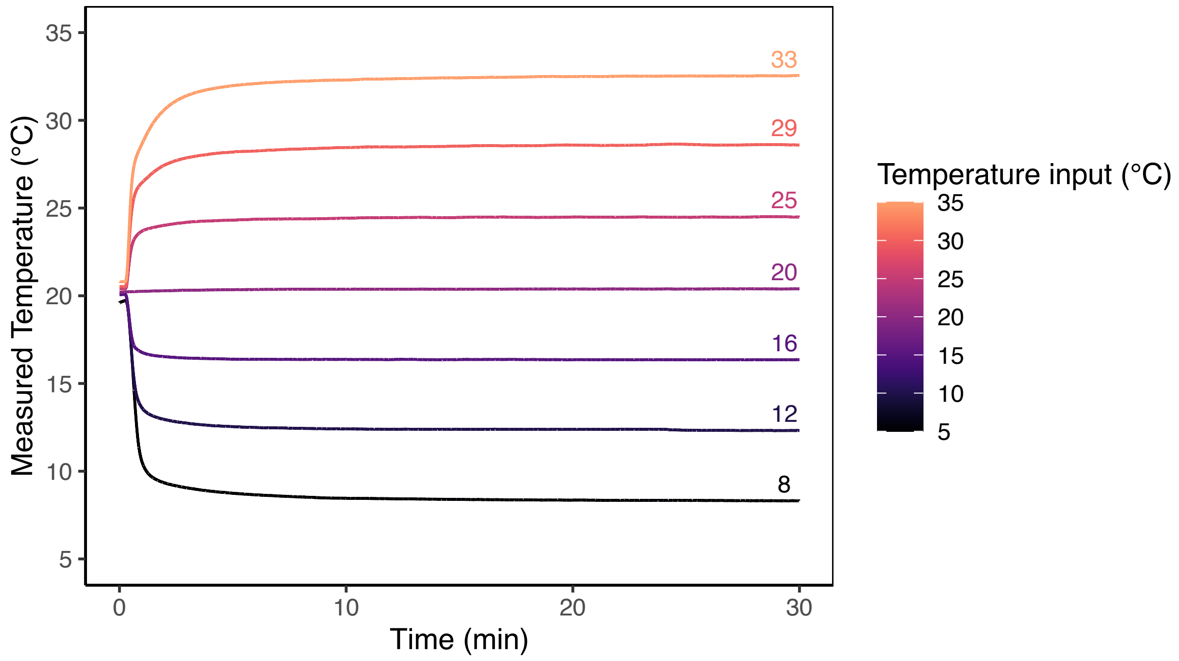

Fig. S1. Representative measurement of the effective temperature within the first 30 min of acclimation in the Phenoplate setup. 250 µL of control sample (f/2 medium) were placed in the setup at control temperature (20°C) under ambient light. Contextually, the measuring end of a Teflon-coated temperature sensor (TSUB21, FireSting-O2, Pyroscience, Germany) was inserted in the sample, spanning for the whole length of the well. After the sensor was in place, we initiated the measurements: the sample was maintained at 20°C for 10 s, after which it was switched to the desired input temperature (5-35°C). The temperature acclimation was followed for 30 min, corresponding to the pre-treatment performed in all our experiments. Color represents the input temperature on the thermocycler, while data points represent the measured values. The numbers at the end of each trace represent the measured temperature reached after 30 min of incubation.

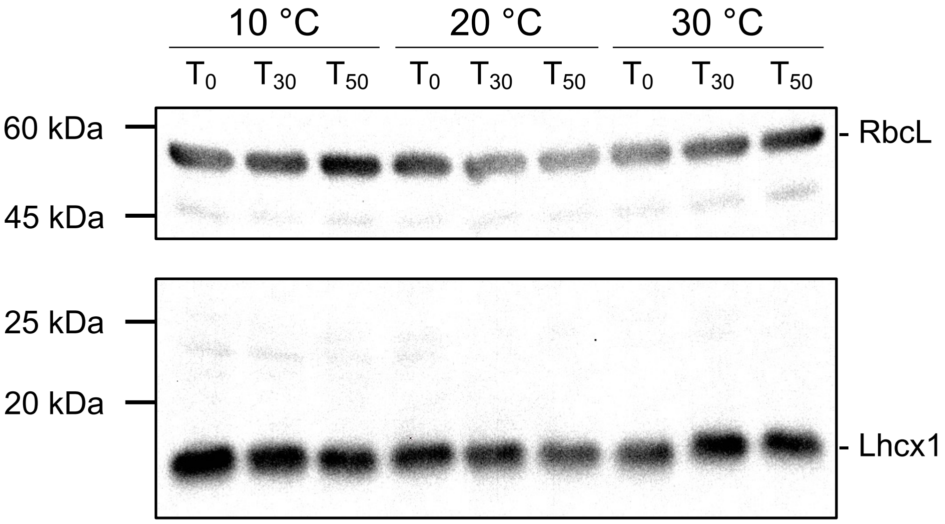

Fig. S2. Western Blot for determination of Lhcx1 content, following temperature stress experiment on wt *P. tricornutum*. After blotting, the upper half of the membrane was incubated with a Rubisco antibody (Anti-RbcL) and the lower half with an antibody detecting all four Lhcx isoforms (Anti-Lhcx). Bands corresponding to rubisco (RbcL) and Lhcx1 (the main Lhcx isoform of *P. tricornutum*) are indicated on the right. On the top (from left to right) samples represent protein extracts (1 µg chlorophyll *a* per sample) before application of temperature stress (T_0), after 30 min (T_{30}), and after 5 min of strong actinic light induction (~700 µmol photons m^{-2} s^{-1}) plus 15 min recovery in low light (T_{50}) at 10, 20 and 30°C, respectively.

Table S1. Comparison of light intensities measured with an integrating sphere or planar sensor in air. Growth chamber: culture conditions used in this study. **IMAGING-PAM (AL)**: actinic light steps displayed by our IMAGING-PAM unit. **PAR sphere**: measured with the spherical sensor of a ULM-500 light meter (Heinz Walz, Germany). **PAR planar**: measured with the planar sensor of a LI-185B Quantum Radiometer Photometer (LI-COR inc., USA). All PAR units are expressed in µmol photons m^{-2} s^{-1}.

	**PAR sphere**	**PAR planar**
**Growth chamber**		
Culture collection, 16°C	~10	~5
Shaking cultures, 20°C	30	20
**IMAGING-PAM (AL)**		
1	12	5.5
2	28	15
3	44	22
4	60	30
6	95	48
8	129	65
10	180	91
11	232	120
12	301	160
13	388	200
14	509	260
15	645	330

Table S2. Results of Linear Mixed-Effect Model (LMM) analysis on NPQ induction-recovery assays. YII and NPQ kinetics data were divided in three groups, corresponding to the three phases of the experiment: acclimation, induction and recovery (**Fig. 1**). For each group, we tested the effect of temperature and added the random effect of replicates (accounting for repeated measures). The test was performed for each line separately, setting 20°C as control. p-values were computed from the t-statistics using Satterthwaite's approximation for degrees of freedom. Asterisks indicate the statistical significance of each temperature condition against the control (20°C): *: $p < 0.05$; **: $p < 0.005$, *** $p < 0.005$.

Phase		T (°C)	p-value					
			vde KO		wt		*zep3 KO*	
**Acclimation (-30–0 min)**	**YII**	5	0.5026		0.0246	*	0.1871	
		10	0.5919		0.0261	*	0.3159	
		15	0.8241		0.2149		0.5816	
		25	0.8437		0.2120		0.2514	
		30	0.3236		0.5433		0.3278	
		35	0.9894		0.0450	*	0.0055	**
	**NPQ**	5	0.1589		0.5221		0.5401	
		10	0.2412		0.5160		0.4165	
		15	0.3489		0.6988		0.4748	
		25	0.9790		0.8217		0.8807	
		30	0.3611		0.3282		0.2109	
		35	0.0021	**	0.0006	***	0.0008	***
**Induction (0–5 min)**	**YII**	5	4.02e-19	***	1.34e-22	***	4.62e-26	***
		10	2.12e-10	***	1.29e-11	***	8.68e-15	***
		15	0.0024	**	0.0001	***	3.69e-05	***
		25	1.52e-12	***	1.06e-14	***	1.65e-16	***
		30	4.83e-09	***	9.62e-10	***	1.15e-10	***
		35	0.9839		0.9604		0.8991	
	**NPQ**	5	1.94e-07	***	3.02e-07	***	2.35e-08	***
		10	8.48e-06	***	0.0003	***	9.26e-06	***
		15	1.75e-06	***	0.1276		0.0318	*
		25	0.3886		0.0718		0.1130	
		30	0.0024	**	0.0066	**	0.0004	***
		35	5.93e-54	***	2.11e-08	***	4.84e-10	***
**Recovery (5–20 min)**	**YII**	5	1.22e-40	***	1.38e-52	***	3.69e-12	***
		10	0.0140	*	0.1173		0.1068	
		15	9.06e-12	***	0.5466		0.9168	
		25	3.31e-17	***	0.0530		0.0006	***
		30	3.65e-17	***	2.47e-31	***	9.07e-13	***
		35	4.18e-25	***	2.3e-100	***	4.0e-119	***
	**NPQ**	5	2.63e-45	***	0.0004	***	9.6e-157	***
		10	7.68e-25	***	0.0093	**	3.2e-122	***
		15	1.25e-22	***	0.0946		1.5e-45	***
		25	6.96e-13	***	0.0016	**	0.3650	
		30	0.0004	***	1.01e-27	***	8.46e-95	***
		35	2.5e-225	***	2.7e-154	***	1.9e-210	***

Table S3. Results of multiple comparison t-test on pigment data from induction-recovery assays (Fig. 3). For each time point, asterisks indicate the statistical significance of each temperature condition tested against the control (20°C), according to adjusted p-value of multiple comparison t-test: *: p < 0.05 ; **: p < 0.005, *** p < 0.005.

Pigment	T (°C)	p-value (adj.)					
		-30 min	0 min	5 min		20 min	
**Dd**	5	1	1	0.0102	*	0.00213	**
	10	1	1	0.208		0.154	
	15	1	1	0.653		0.652	
	25	1	1	0.653		0.852	
	30	1	1	0.0639		0.0549	
	35	1	0.109	2.23e-05	***	1.59e-08	***
**Dt**	5	NA	1	0.00891	**	0.000933	***
	10	NA	1	0.0972		0.324	
	15	NA	1	0.409		1	
	25	NA	1	0.581		1	
	30	NA	1	0.0754		4.71e-05	***
	35	NA	0.141	0.00129	**	4.95e-11	***
**Dd + Dt**	5	1	1	1		0.0404	*
	10	1	1	1		0.364	
	15	1	1	1		0.858	
	25	1	1	1		1	
	30	1	1	1		1	
	35	1	0.546	0.693		0.00221	**

Table S4. Results of Linear Mixed-Effect Model (LMM) analysis on light curve assays. YII and NPQ kinetics data was tested in a linear model accounting for the effect of temperature, culture, phase of the experiment (acclimation, light curve and recovery) and replicates (random, accounting for repeated measures). The test was performed for on the whole dataset, setting 20°C and wt as controls. p-values were computed from the t-statistics using Satterthwaite's approximation for degrees of freedom. Asterisks indicate the statistical significance of each term (temperature or culture) against the control (20 °C or wt): *: p < 0.05 ; **: p < 0.005, *** p < 0.005.

	**Term (T °C or Culture)**	**p-value**	
**YII**	5	2.59e-10	***
	10	0.0005	***
	15	0.1244	
	25	0.2127	
	30	0.5218	
	vde KO	0.0164	*
	zep3 KO	0.5964	
**NPQ**	5	0.0429	*
	10	0.1468	
	15	0.4325	
	25	0.9347	
	30	2.00e-06	***
	vde KO	3.75e-67	***
	zep3 KO	5.95e-06	***
